# Prevalence of neurodevelopmental disorders among US children and adolescents in 2019 and 2020

**DOI:** 10.3389/fpsyg.2022.997648

**Published:** 2022-11-24

**Authors:** Yiwei Yang, Shi Zhao, Meihui Zhang, Mi Xiang, Jian Zhao, Shucheng Chen, Hui Wang, Lefei Han, Jinjun Ran

**Affiliations:** ^1^School of Public Health, Shanghai Jiao Tong University School of Medicine, Shanghai, China; ^2^JC School of Public Health and Primary Care, Chinese University of Hong Kong, Hong Kong, Hong Kong SAR, China; ^3^MOE-Shanghai Key Laboratory of Children’s Environmental Health, Xinhua Hospital, Shanghai Jiao Tong University School of Medicine, Shanghai, China; ^4^School of Nursing, The Hong Kong Polytechnic University, Hong Kong, Hong Kong SAR, China; ^5^School of Global Health, Chinese Center for Tropical Diseases Research, Shanghai Jiao Tong University School of Medicine, Shanghai, China

**Keywords:** neurodevelopmental disorders, attention-deficit/hyperactivity disorder, autism spectrum disorder, nationwide prevalence, United States

## Abstract

**Background:**

Concerning the changes in the prevalence of neurodevelopmental disorders (NDDs), we estimate the prevalence of attention-deficit/hyperactivity disorder (ADHD), autism spectrum disorder (ASD), intellectual disorder (ID), and learning disability (LD) among US children and adolescents aged 3–17 years in 2019 and 2020.

**Methods:**

The study includes 14,983 US children and adolescents aged 3–17 years in 2019 and 2020 from the National Health Interview Survey (NHIS). Parents were interviewed about whether their children ever and/or currently had NDDs diagnosed. Prevalence estimates of NDDs were calculated with a survey-based weighting scheme. Logistic regression models were used to estimate the associations between NDDs prevalence and subgroups.

**Results:**

The weighted prevalence of ADHD, ASD, ID, and LD was 8.5% (95% CI: 7.9–9.2%), 2.9% (95% CI: 2.6–3.4%), 1.4% (95% CI: 1.2–1.7%), and 6.4% (95% CI: 5.8–7.0%), respectively. A higher prevalence of ADHD, ASD, ID, and LD was observed in boys, those who ever had anxiety or depression symptoms, those with lower family income, those living in a rented house, ever been bullied, and ever lived with anyone mentally ill.

**Conclusion:**

The study found the prevalence of ADHD, ASD, ID, and LD was different by demographics, comorbidity/mental problems, household/parental characteristics, and stressful life events.

## Introduction

Children and adolescent neurodevelopmental disorders (NDDs), including attention-deficit/hyperactivity disorder (ADHD), autism spectrum disorder (ASD), intellectual disorder (ID), learning disability (LD), and others, may result in serious delay or irregularity in growth, especially functional, structural, and cognitive maturation (Thapar et al., 2017; Antshel and Russo, 2019; Nickel et al., 2019). Belated diagnosis and treatments of NDDs would predispose life-long disabilities to individuals and bring heavy burdens to families and society (Lai et al., 2014; Posner et al., 2020). In addition, children with NDDs are likely to be diagnosed with various mental or psychiatric problems in adulthood, which may lead to extreme events or secondary negative impacts on individuals, families, and society (Du Rietz et al., 2021).

Continued monitoring of the prevalence of NDDs in children and adolescents is necessary to evaluate the current disease burdens and predict future impacts. According to the early evidence before 2018, the prevalence of ADHD and ASD was steadily rising among US children and adolescents (Xu et al., 2018; Zablotsky et al., 2019a; Zablotsky and Black, 2020). The observed increase may partly attribute to the improved perception and alternative diagnosis methods for ADHD and ASD, while unknown factors cannot be ignored and warrant further investigation (Lai et al., 2014; Posner et al., 2020). The trends in the prevalence of ID and LD seemed to plateau in the last decades (Zablotsky et al., 2019a). However, the NDDs prevalence may be overestimated because previous studies in US children and adolescents counted cases by ever-diagnosed NDDs rather than currently had NDDs, which might mix up with those who were ever diagnosed with NDDs but no longer meet the criteria. Recent studies showed that NDD symptoms could be reduced to a non-clinical level by early non-pharmaceutical or pharmaceutical interventions (Cramer et al., 2011; Berry-Kravis et al., 2018). Prevalence based on those who currently have NDDs rather than ever had NDDs would mirror the accurate level (Kogan et al., 2018).

Interactions between genetic heritability and environmental factors contribute to the onset of children and adolescent NDDs (Bourgeron, 2015; Kasparek et al., 2015). Previous studies focused more on the prevalent distribution of demography and socioeconomic status (Kogan et al., 2018; Xu et al., 2018; Zablotsky et al., 2019a). Few studies investigated the potential entanglement of NDDs with stressful life events, comorbidity/psychiatric symptoms, and household/parental characteristics. Although cross-sectional studies are limited in drawing causal inferences, this study attempted to detail the prevalence of NDDs on more population characteristics among US children and adolescents to underly diversified materials for risk factors.

## Materials and methods

### Data source

We retrieved data from the National Health Interview Survey (NHIS) in 2019 and 2020. NHIS, conducted by the National Center for Health Statistics (NCHS), is an annual, nationally representative, multistage-sampling interview survey to collect health-related information on the civilian non-institutionalized US population (National Center for Health Statistics, 2020, 2021). NHIS was approved by the research ethics review board of the NCHS and has been widely used to estimate the nationwide prevalence of various diseases in the US (Xu et al., 2018; Gu et al., 2021). Compared with previous versions, NHIS in 2019 and 2020 was redesigned to minimize respondent burden and enhance data quality. During the sampling procedure of NHIS, one sample adult (≥18 years old) and one sample child (≤17 years old, if any children live in the household) were randomly selected from each household. Information about the sample child was collected from a parent or adult who was knowledgeable about and responsible for the healthcare of the sample child. This study only retrieved children’s data for analysis. A total of 9,193 and 5,790 interviewed sample child questionnaires have been collected in 2019 and 2020, respectively, and the corresponding final sample child response rates were approximately 59.1 and 47.8%. The weighting process for 2019 and 2020 was also updated since multilevel regression models, including predictive variables of both survey response and key health outcomes, were used for non-response adjustment.

### Data collection

The NDD outcomes were ascertained by asking the parents of sampled children whether their children ever had ADHD, ASD, ID, or LD told by a representative of a school or a health professional. The parents were also questioned if their child currently had these NDDs. Information on demographics, comorbidities, or psychological disorders, as well as household or parental factors, were collected and grouped. The stratification included age (3–11 and 12–17 years), sex (boy and girl), race/ethnicity (non-Hispanic white, non-Hispanic black, Hispanic, and non-Hispanic other and multiple races), health insurance (uninsured, private only, and any public), urbanicity (urban and rural), geographic region (Northeast, Midwest, South, and West), ever had asthma (yes and no), ever had anxiety symptoms (yes and no), ever had depressive symptoms (yes and no), household structure (single parent, married parents, and others), number of children (1 child and ≥ 2 children), living with an elder/elders (yes and no), the highest level of education (below high school, high school, and beyond high school), family income to poverty ratio (IPR, grouped into < 100, 100–199, 200–399, and ≥ 400%), and home ownership (owned/being bought and rented/other arrangements). Questions of ever had anxiety symptoms and ever had depression symptoms only include children and adolescents aged 5–17 years old. Stressful life events of the child or his/her family members were included in the dataset of 2019 but unavailable for 2020. Relevant information included experience as a victim of/witnessed violence (yes and no), bullied by others (yes and no), ever lived with a parent who was incarcerated (yes and no), ever lived with anyone mentally ill (yes and no), and ever lived with anyone with an alcohol problem (yes and no).

### Statistical analysis

Prevalence estimates of NDDs were stratified by demographics, stressful life events, comorbidities or psychological disorders, and household or parental factors. Prevalence estimates with corresponding 95% confidence intervals (95% CIs) of ADHD, ASD, ID, and LD were weighted using survey procedures by “*survey*” and “*srvyr*” packages in the R environment (version 3.6.1). The non-response weights were calculated by controlling for age, sex, race/ethnicity, education level, census division, and Metropolitan Statistical Area status. The 95% CI was asymmetric about the point estimate due to the logit transformation. The *P*-values showing the statistical difference across strata were calculated by the Rao-Scott χ^2^-test with adjusted *F*-statistic, considering the complex sample design. Multivariable logistic regression models were used to estimate the associations between NDDs prevalence and the subgroups after controlling for age, sex, race, health insurance, urbanicity, and geographic regions, considering the various potential confounders. Statistical significance was claimed when *P*-value < 0.05.

## Results

**FIGURE 1:**
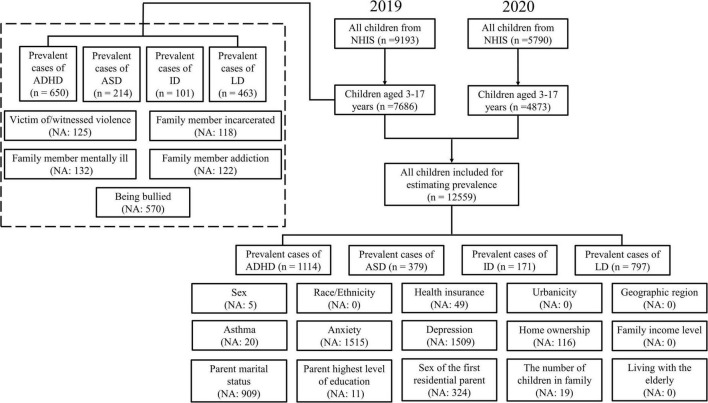
A flowchart of children and adolescents included in the study and the number of missing values for each variable.

Based on the NHIS data in 2019 and 2020, the estimated population of 61.3 million children and adolescents aged over 3–17 years can be represented in the analysis, where we identified 5.2 million individuals with ADHD, 1.8 million with ASD, 0.9 million with ID, and 3.9 million with LD. Data filtering and the unweighted number of NDDs are shown in Figure 1. The weighted prevalence of currently had NDDs was 8.5% (95% CI: 7.9–9.2%) for ADHD, 2.9% (95% CI: 2.6–3.4%) for ASD, 1.4% (95% CI: 1.2–1.7%) for ID, and 6.4% (95% CI: 5.8–7.0%) for LD (Table 1). Boys had a weighted prevalence of 11.1% (95% CI: 10.2–12.4%) for ADHD and 4.4% (95% CI: 3.8–5.0%) for ASD, compared with 5.8% (95% CI: 5.0–6.6%) for ADHD and 1.4% (95% CI: 1.1–1.9%) for ASD in girls. Higher prevalence of ID and LD were also observed in boys than girls. The prevalence of ADHD and ASD was higher among those who ever had anxiety than those without anxiety (ADHD among those with anxiety 14.8%, 95% CI: 13.5–16.1% vs. ADHD among those without anxiety 5.1%, 95% CI: 4.4–5.9%; ASD among those with anxiety 5.2%, 95% CI: 4.4–6.1% vs. ASD among those without anxiety 1.3%, 95% CI: 0.9–1.8%). Similarly, ADHD and ASD were more prevalent among those who ever had depression than those not (ADHD with depression 15.9%, 95% CI: 14.5–17.5% vs. ADHD without depression 7.1%, 95% CI: 6.3–7.9%; ASD with depression 4.9, 95% CI: 4.0–6.0% vs. ASD without depression 2.4%, 95% CI: 2.0–2.8%). The weighted prevalence of ID and LD showed similar patterns in anxiety and depression. Higher prevalence of ADHD, ASD, ID, and LD were also shown among children or adolescents who lived in rented houses. In addition, the prevalence of ADHD and LD was higher among those without health insurance (ADHD 8.7%, 95% CI: 8.0–9.4%; LD 6.6%, 95% CI: 6.0–7.2%), ever had asthma (ADHD 13.4%, 95% CI: 11.5–15.5%; LD 11.5%, 95% CI: 9.6–13.7%), living with a single parent (ADHD 11.7%, 95% CI: 10.3–13.4%; LD 8.3%, 95% CI: 7.0–9.7%), and family income to poverty ratio less than 100% (ADHD 11.8%, 95% CI: 9.9–14.0%; LD 11.0%, 95% CI: 9.2–13.0%) (Table 1).

**TABLE 1:** Prevalence of cause-specific neurodevelopmental disorders among US children and adolescents aged 3–17 years in 2019 and 2020^a^.

	ADHD		ASD		ID		LD	
				
	Prevalence, % (95% CI)^b^	*P*-value^c^	Prevalence, % (95% CI)^b^	*P*-value^c^	Prevalence, % (95% CI)^b^	*P*-value^c^	Prevalence, % (95% CI)^b^	*P*-value^c^
Total number of prevalent cases, *n*	5.2 million		1.8 million		0.9 million		3.9 million	
**Overall**								
Current diagnosed	8.5 (7.9, 9.2)		2.9 (2.6, 3.4)		1.4 (1.2, 1.7)		6.4 (5.8, 7.0)	
Ever diagnosed	9.5 (8.8, 10.2)		3.1 (2.8, 3.6)		1.9 (1.7, 2.3)		7.6 (7.0, 8.2)	
**Child demographic**								
**Age, years**								
3–11	6.6 (5.8, 7.4)	< 0.001	2.9 (2.4, 3.4)	0.77	1.3 (1.0, 1.7)	0.20	5.3 (4.7, 6.1)	< 0.001
12–17	11.3 (10.2, 12.4)		3.0 (2.4, 3.7)		1.7 (1.3, 2.1)		7.9 (6.9, 8.9)	
**Sex**								
Girls	5.8 (5.0, 6.6)	< 0.001	1.4 (1.1, 1.9)	< 0.001	1.1 (0.8, 1.5)	0.02	5.0 (4.3, 5.7)	< 0.001
Boys	11.1 (10.2, 12.1)		4.4 (3.8, 5.0)		1.8 (1.4, 2.3)		7.7 (6.9, 8.6)	
**Race/ethnicity**								
Non-Hispanic white	10.2 (9.2, 11.2)	< 0.001	3.0 (2.5, 3.5)	0.96	1.4 (1.1, 1.8)	0.31	6.4 (5.7, 7.3)	0.002
Non-Hispanic black	7.8 (6.2, 9.9)		3.1 (1.9, 5.0)		2.0 (1.1, 3.5)		8.6 (6.7, 10.8)	
Hispanic	6.4 (5.4, 7.7)		2.8 (2.1, 3.6)		1.4 (1.0, 2.1)		6.0 (5.1, 7.2)	
Non-Hispanic other and multiple race	6.2 (4.7, 8.0)		2.8 (1.9, 4.1)		0.8 (0.4, 1.6)		4.0 (2.9, 5.4)	
**Health insurance^d^**								
Uninsured	8.7 (8.0, 9.4)	0.03	3.0 (2.6, 3.4)	0.22	1.5 (1.2, 1.8)	0.17	6.6 (6.0, 7.2)	0.001
Insured	5.5 (3.6, 8.3)		1.9 (0.9, 3.9)		0.7 (0.2, 2.1)		3.1 (1.9, 5.0)	
**Urbanicity**								
Urban^e^	8.1 (7.5, 8.8)	0.02	3.1 (2.7, 3.5)	0.13	1.5 (1.2, 1.8)	0.36	6.2 (5.6, 6.9)	0.12
Rural	10.9 (8.6, 13.7)		2.1 (1.3, 3.4)		1.1 (0.6, 2.0)		7.4 (6.0, 9.1)	
**Geographic region**								
Northeast	8.4 (7.0, 10.1)	0.001	3.7 (2.8, 5.0)	0.14	2.3 (1.6, 3.4)	0.06	7.2 (5.9, 8.7)	0.50
Midwest	9.5 (7.7, 11.5)		3.4 (2.5, 4.6)		1.2 (0.8, 2.0)		6.0 (4.8, 7.5)	
South	9.6 (8.6, 10.7)		2.5 (2.1, 3.0)		1.2 (0.9, 1.6)		6.6 (5.7, 7.5)	
West	6.1 (5.1, 7.3)		2.7 (2.0, 3.7)		1.4 (0.9, 2.1)		5.9 (4.8, 7.2)	
**Comorbidity**								
**Asthma**								
Yes	13.4 (11.5, 15.5)	< 0.001	4.0 (3.0, 5.3)	0.02	1.9 (1.2, 2.9)	0.20	11.5 (9.6, 13.7)	< 0.001
No	7.9 (7.2, 8.6)		2.8 (2.4, 3.2)		1.4 (1.1, 1.7)		5.7 (5.2, 6.3)	
**Anxiety^f^**								
Yes	14.8 (13.5, 16.1)	< 0.001	5.2 (4.4, 6.1)	< 0.001	2.5 (2, 3.1.0)	< 0.001	10.7 (9.5, 11.9)	< 0.001
No	5.1 (4.4, 5.9)		1.3 (0.9, 1.8)		0.7 (0.4, 1.0)		3.8 (3.2, 4.5)	
**Depression^g^**								
Yes	15.9 (14.5, 17.5)	< 0.001	4.9 (4.0, 6.0)	< 0.001	2.7 (2.0, 3.6)	< 0.001	11.7 (10.2, 13.3)	< 0.001
No	7.1 (6.3, 7.9)		2.4 (2.0, 2.8)		1.0 (0.8, 1.4)		5.0 (4.4, 5.6)	
**Parent characteristics**								
**Marital status**								
Married	6.6 (5.8, 7.4)	< 0.001	2.5 (2.2, 3.0)	0.002	1.2 (0.9, 1.7)	0.14	5.2 (4.5, 5.9)	< 0.001
Single	11.7 (10.3, 13.4)		4.2 (3.3, 5.3)		1.9 (1.4, 2.6)		8.3 (7.0, 9.7)	
Other^h^	10.8 (8.7, 13.2)		2.8 (1.9, 4.1)		1.8 (1.0, 3.0)		8.2 (6.4, 10.4)	
**Highest level of education^i^**								
Below high school	7.5 (5.2, 10.8)	0.65	2.3 (1.1, 4.8)	0.75	2.5 (1.4, 4.5)	0.05	8.3 (5.9, 11.6)	0.01
High school	9.0 (7.5, 10.7)		3.0 (2.3, 4.0)		1.8 (1.1, 2.7)		7.9 (6.5, 9.6)	
Beyond high school	8.5 (7.8, 9.3)		3.0 (2.6, 3.4)		1.3 (1.0, 1.6)		5.9 (5.3, 6.5)	
**Family characteristics**								
**The number of children**								
One child	9.5 (8.6, 10.5)	0.05	3.6 (3.1, 4.3)	0.02	1.5 (1.2, 2.0)	0.61	6.7 (6.0, 7.6)	0.40
≥2 children	8.3 (7.5, 9.1)		2.8 (2.3, 3.3)		1.4 (1.1, 1.8)		6.3 (5.6, 7.0)	
**Living with the elderly^j^**								
No	8.4 (7.7, 9.1)	0.13	2.9 (2.5, 3.3)	0.15	1.4 (1.1, 1.7)	0.26	6.2 (5.7, 6.9)	0.06
Yes	10.1 (8.0, 12.6)		3.8 (2.6, 5.5)		2.0 (1.1, 3.6)		8.2 (6.3, 10.7)	
**Family income to poverty ratio**								
<100%	11.8 (9.9, 14.0)	< 0.001	4.6 (3.4, 6.3)	0.001	1.8 (1.1, 2.8)	0.03	11.0 (9.2, 13.0)	< 0.001
100–199%	9.3 (8.0, 10.8)		2.6 (1.9, 3.5)		2.0 (1.4, 3.0)		6.7 (5.6, 8.2)	
200–399%	7.1 (6.2, 8.1)		3.1 (2.5, 3.8)		1.1 (0.8, 1.6)		4.8 (4.1, 5.7)	
≥400%	7.6 (6.7, 8.7)		2.2 (1.7, 2.8)		1.1 (0.8, 1.5)		5.1 (4.3, 6.1)	
**Homeownership**								
Rented house or other	9.4 (8.3, 10.7)	0.03	3.6 (2.9, 4.5)	0.01	2.0 (1.5, 2.6)	0.006	7.8 (6.7, 8.9)	< 0.001
Owned house	8.0 (7.2, 8.8)		2.6 (2.2, 3.0)		1.1 (0.9, 1.5)		5.5 (4.9, 6.2)	

ADHD, attention deficit hyperactivity disorder; ASD, autism spectrum disorder; ID, intellectual disorder; LD, learning disorder.

^a^In 2019 and 2020, 14,975 US children (aged 3–17 years old) were included in the analysis. Information on ADHD, ASD, ID, and LD was collected from parent reports. Respondents were asked whether a doctor or other healthcare professional ever told them that the child had ADHD, ASD, ID, and LD, and whether the sample child currently had this developmental condition.

^b^All estimates were weighted, and 95% of CIs were based on a logit transformation and might be asymmetric about the point estimate.

^c^*P*-values across the stratum was calculated from the Rao-Scott χ^2^-test with an adjusted *F*-statistic.

^d^Individuals are considered currently insured if they currently have coverage through private health insurance, Medicare, Medicaid, Children’s Health Insurance Program (CHIP), military [TRICARE, Veterans Administration (VA), and CHAMP-VA], other state-sponsored health plans, or other government programs. Individuals without any of the aforementioned coverages or with only Indian Health Service coverage or a non-comprehensive plan that covers only dental, vision, or prescription drugs are considered uninsured.

^e^Urban area stands for large central metro, large fringe metro, medium metro, and small metro.

^f^Individuals are considered to have anxiety symptoms if they seemed anxious, nervous, or worried equal to or more than a few times a year.

^g^Individuals are considered to have depression symptoms if they seemed sad or depressed equal to or more than a few times a year.

^h^Other means cohabiting parents residing in the same household as sample children or at least one related or unrelated adult (not a parent) in the same household as sample children.

^i^The highest level of education refers to the highest level of education among all sample child’s parents.

^j^Individuals are considered to live with the elderly when there is at least one person aged over 65 in the family.

The prevalence of NDDs among US children and adolescents was different from their stressful life experiences. We found a higher prevalence of ADHD, ASD, ID, and LD among those who were the victim of/witnessed violence (ADHD 20.3%, 95% CI: 16.4–24.9%; ASD 3.7%, 95% CI: 2.1–6.4%; ID 3.5%, 95% CI: 2.0–6.1%; LD 14.8%, 95% CI: 11.3–19.1%), being bullied by others (ADHD 18.2%, 95% CI: 15.7–21.0%; ASD 6.6%, 95% CI: 5.0–8.7%; ID 2.5%, 95% CI: 1.6–4.1%; LD 14.8%, 95% CI: 12.4–17.5%), and ever lived with anyone mentally ill (ADHD 20.0%, 95% CI: 16.6–23.9%; ASD 4.4%, 95% CI: 2.9–6.6%; ID 3.6%, 95% CI: 2.3–5.4%; LD 12.6%, 95% CI: 10.0–15.7%) (Table 2). Higher prevalence of ADHD (18.0%, 95% CI: 14.3–22.3%), ID (2.77%, 95% CI: 1.47, 5.21), and LD (11.4%, 95% CI: 8.8–14.7%) were also found among individuals with incarcerated family members. Using logistic regression models, we found that the prevalence of ADHD, ASD, ID, and LD was all associated with sex, psychological problems, family income levels, homeownership, and stressful life events, including ever being a victim of/witnessed violence (except for ASD), bullied by others, ever lived with a parent who was incarcerated (except for ASD), ever lived with anyone mentally ill, and ever lived with anyone addicting to alcohol/drug. In addition, children and adolescents without health insurance, ever had asthma, living with a single parent, or others have higher risks for ADHD and LD (Table 3).

**TABLE 2:** Subgroup prevalence by stressful life events among US children and adolescents aged 3–17 years in 2019^a^.

	ADHD		ASD		ID		LD	
				
	Prevalence, % (95% CI)^b^	*P*-value^c^	Prevalence, % (95% CI) ^b^	*P*-value^c^	Prevalence, % (95% CI) ^b^	*P*-value^c^	Prevalence, % (95% CI) ^b^	*P*-value^c^
**Victim of/witnessed violence**								
Yes	20.3 (16.4, 24.9)	< 0.001	3.7 (2.1, 6.4)	0.19	3.5 (2.0, 6.1)	< 0.001	14.8 (11.3, 19.1)	< 0.001
No	6.7 (6.1, 7.5)		2.5 (2.1, 3.0)		1.1 (0.8, 1.5)		5.0 (4.5, 5.6)	
**Being bullied^d^**								
Yes	18.2 (15.7, 21.0)	< 0.001	6.6 (5.0, 8.7)	< 0.001	2.5 (1.6, 4.1)	0.002	14.8 (12.4, 17.5)	< 0.001
No	6.1 (5.4, 6.8)		1.8 (1.4, 2.2)		1.0 (0.8, 1.4)		4.1 (3.6, 4.7)	
**Family member incarcerated^e^**								
Yes	18.0 (14.3, 22.3)	< 0.001	3.1 (1.8, 5.4)	0.49	3.0 (1.8, 5.2)	0.001	11.4 (8.8, 14.7)	< 0.001
No	6.8 (6.2, 7.6)		2.5 (2.1, 3.0)		1.1 (0.9, 1.5)		5.2 (4.6, 5.8)	
**Family member mentally ill^f^**								
Yes	20.0 (16.6, 23.9)	< 0.001	4.4 (2.9, 6.6)	0.006	3.6 (2.3, 5.4)	< 0.001	12.6 (10.0, 15.7)	< 0.001
No	6.4 (5.8, 7.1)		2.4 (2.0, 2.9)		1.0 (0.8, 1.4)		4.9 (4.4, 5.5)	
**Family member addiction^g^**								
Yes	15.6 (12.9, 18.6)	< 0.001	3.4 (2.1, 5.3)	0.24	2.2 (1.3, 3.7)	0.03	9.3 (7.2, 11.9)	< 0.001
No	6.8 (6.1, 7.5)		2.5 (2.1, 3.0)		1.1 (0.9, 1.5)		5.2 (4.6, 5.9)	

ADHD, attention deficit hyperactivity disorder; ASD, autism spectrum disorder; ID, intellectual disorder; LD, learning disorder.

^a^Information on ADHD, ASD, ID, and LD was collected from parent reports. Respondents were asked whether a doctor or other healthcare professional ever told them that the child had ADHD, ASD, ID, and LD, and whether the sample child currently had this developmental condition. The personal experience of the child or his/her family members was included in the dataset of 2019 but unavailable for 2020.

^b^All estimates were weighted, and 95% of CIs were based on a logit transformation and might be asymmetric about the point estimate.

^c^*P*-values across the stratum was calculated from the Rao-Scott χ^2^-test with an adjusted *F*-statistic.

^d^Individuals are considered being bullied when parents are certainly true or somewhat true that children have been picked on or bullied by others in the last 6 months.

^e^Individuals are considered to have a family member who was incarcerated when they had ever lived with a parent who was incarcerated.

^f^Individuals are considered to have a family member who was mentally ill when they had ever lived with anyone mentally ill/severely depressed.

^g^Individuals are considered to have a family member with addiction problems when they had ever lived with anyone with alcohol/drug problems.

**TABLE 3:** Weighted logistic regression models for ADHD, ASD, ID, and LD among US children and adolescents aged 3–17 years^a^.

	ADHD		ASD		ID		LD	
				
	Adjusted odds ratio (95% CI)^b^	*P*-value	Adjusted odds ratio (95% CI)^b^	*P*-value	Adjusted odds ratio (95% CI)^b^	*P*-value	Adjusted odds ratio (95% CI)^b^	*P*-value
**Child demographic**								
**Age, year**								
3–11	1.00 [Reference]		1.00 [Reference]		1.00 [Reference]		1.00 [Reference]	
12–17	1.80 (1.52, 2.13)	< 0.001	1.05 (0.79, 1.40)	0.72	1.31 (0.87, 1.97)	0.19	1.52 (1.25, 1.85)	< 0.001
**Sex**								
Women	1.00 [Reference]		1.00 [Reference]		1.00 [Reference]		1.00 [Reference]	
Men	2.07 (1.76, 2.45)	< 0.001	3.11 (2.29, 4.23)	< 0.001	1.62 (1.09, 2.4)	0.02	1.61 (1.34, 1.94)	< 0.001
**Race/ethnicity**								
Non-Hispanic white	1.00 [Reference]		1.00 [Reference]		1.00 [Reference]		1.00 [Reference]	
Non-Hispanic black	0.71 (0.53, 0.94)	0.02	1.10 (0.63, 1.90)	0.74	1.54 (0.75, 3.17)	0.24	1.40 (1.02, 1.92)	0.04
Hispanic	0.67 (0.54, 0.84)	< 0.001	0.97 (0.69, 1.36)	0.85	1.08 (0.64, 1.81)	0.77	1.00 (0.79, 1.26)	0.98
Non-Hispanic other and multiple race	0.63 (0.47, 0.86)	0.003	0.96 (0.63, 1.46)	0.85	0.59 (0.28, 1.23)	0.16	0.63 (0.44, 0.88)	0.008
**Health insurance^c^**								
Uninsured	1.00 [Reference]		1.00 [Reference]		1.00 [Reference]		1.00 [Reference]	
Insured	1.74 (1.09, 2.78)	0.02	1.56 (0.73, 3.33)	0.26	2.11 (0.65, 6.80)	0.21	2.30 (1.38, 3.84)	0.001
**Urbanicity^d^**								
Urban	1.00 [Reference]		1.00 [Reference]		1.00 [Reference]		1.00 [Reference]	
Rural	1.23 (0.93, 1.63)	0.14	0.7 (0.43, 1.15)	0.16	0.83 (0.43, 1.61)	0.59	1.26 (0.99, 1.62)	0.07
**Geographic region**								
Northeast	1.00 [Reference]		1.00 [Reference]		1.00 [Reference]		1.00 [Reference]	
Midwest	1.07 (0.80, 1.44)	0.65	0.95 (0.60, 1.49)	0.81	0.55 (0.30, 1.02)	0.06	0.80 (0.58, 1.10)	0.17
South	1.21 (0.95, 1.54)	0.12	0.68 (0.47, 0.98)	0.04	0.51 (0.30, 0.86)	0.01	0.88 (0.67, 1.14)	0.32
West	0.76 (0.58, 1.01)	0.06	0.74 (0.47, 1.15)	0.18	0.61 (0.32, 1.15)	0.13	0.86 (0.62, 1.18)	0.34
**Comorbidity**								
**Asthma**								
No	1.00 [Reference]		1.00 [Reference]		1.00 [Reference]		1.00 [Reference]	
Yes	1.61 (1.31, 1.98)	< 0.001	1.31 (0.94, 1.82)	0.11	1.23 (0.75, 2.04)	0.41	1.93 (1.53, 2.42)	< 0.001
**Anxiety^e^**								
No	1.00 [Reference]		1.00 [Reference]		1.00 [Reference]		1.00 [Reference]	
Yes	3.29 (2.72, 3.97)	< 0.001	4.76 (3.26, 6.97)	< 0.001	4.35 (2.60, 7.30)	< 0.001	3.37 (2.67, 4.27)	< 0.001
**Depression^f^**								
No	1.00 [Reference]		1.00 [Reference]		1.00 [Reference]		1.00 [Reference]	
Yes	2.61 (2.22, 3.07)	< 0.001	2.39 (1.80, 3.19)	< 0.001	2.98 (1.94, 4.60)	< 0.001	2.78 (2.26, 3.41)	< 0.001
**Parent characteristics**								
**Marital status**								
Married	1.00 [Reference]		1.00 [Reference]		1.00 [Reference]		1.00 [Reference]	
Single	2.16 (1.76, 2.65)	< 0.001	1.71 (1.23, 2.39)	0.001	1.38 (0.82, 2.32)	0.22	1.55 (1.22, 1.96)	< 0.001
Other^g^	2.02 (1.53, 2.69)	< 0.001	1.19 (0.77, 1.86)	0.43	1.49 (0.81, 2.75)	0.20	1.67 (1.22, 2.30)	0.002
**Highest level of education^h^**								
Below high school	1.00 [Reference]		1.00 [Reference]		1.00 [Reference]		1.00 [Reference]	
High school	1.12 (0.71, 1.77)	0.63	1.31 (0.59, 2.91)	0.50	0.63 (0.30, 1.29)	0.21	0.87 (0.57, 1.33)	0.53
Beyond high school	0.97 (0.64, 1.47)	0.88	1.21 (0.57, 2.56)	0.62	0.43 (0.22, 0.84)	0.01	0.64 (0.43, 0.96)	0.03
**Family characteristics**								
**The number of children**								
One child	1.00 [Reference]		1.00 [Reference]		1.00 [Reference]		1.00 [Reference]	
≥2 children	0.94 (0.80, 1.10)	0.45	0.74 (0.57, 0.95)	0.02	0.96 (0.67, 1.39)	0.84	1.00 (0.84, 1.21)	0.97
**Living with the elderly^i^**								
No	1.00 [Reference]		1.00 [Reference]		1.00 [Reference]		1.00 [Reference]	
Yes	1.25 (0.95, 1.64)	0.11	1.39 (0.93, 2.08)	0.11	1.42 (0.74, 2.70)	0.29	1.31 (0.96, 1.80)	0.09
**Family income to poverty ratio**								
<100%	1.00 [Reference]		1.00 [Reference]		1.00 [Reference]		1.00 [Reference]	
100–199%	0.72 (0.55, 0.93)	0.01	0.52 (0.33, 0.81)	0.004	1.12 (0.61, 2.05)	0.72	0.58 (0.43, 0.77)	< 0.001
200–399%	0.47 (0.37, 0.60)	< 0.001	0.58 (0.39, 0.87)	0.008	0.58 (0.33, 1.05)	0.07	0.39 (0.29, 0.51)	< 0.001
≥400%	0.47 (0.37, 0.60)	< 0.001	0.36 (0.23, 0.56)	< 0.001	0.54 (0.30, 0.95)	0.03	0.39 (0.30, 0.52)	< 0.001
**Homeownership**								
Rented home or other	1.00 [Reference]		1.00 [Reference]		1.00 [Reference]		1.00 [Reference]	
Owned home	0.67 (0.56, 0.81)	< 0.001	0.66 (0.5, 0.87)	0.004	0.57 (0.38, 0.86)	0.007	0.68 (0.55, 0.84)	< 0.001
**Stressful life events**								
**Victim of/witnessed violence**								
No	1.00 [Reference]		1.00 [Reference]		1.00 [Reference]		1.00 [Reference]	
Yes	3.34 (2.51, 4.45)	< 0.001	1.59 (0.84, 3.00)	0.15	3.22 (1.66, 6.25)	0.001	3.10 (2.24, 4.30)	< 0.001
**Being bullied^j^**								
No	1.00 [Reference]		1.00 [Reference]		1.00 [Reference]		1.00 [Reference]	
Yes	3.51 (2.85, 4.31)	< 0.001	3.81 (2.66, 5.46)	< 0.001	2.54 (1.41, 4.57)	0.002	4.22 (3.29, 5.41)	< 0.001
**Family member incarcerated^k^**								
No	1.00 [Reference]		1.00 [Reference]		1.00 [Reference]		1.00 [Reference]	
Yes	2.87 (2.15, 3.84)	< 0.001	1.31 (0.71, 2.41)	0.39	2.77 (1.47, 5.21)	0.002	2.27 (1.66, 3.10)	< 0.001
**Family member mentally ill^l^**								
No	1.00 [Reference]		1.00 [Reference]		1.00 [Reference]		1.00 [Reference]	
x Yes	3.58 (2.78, 4.61)	< 0.001	1.90 (1.21, 2.98)	0.005	3.90 (2.29, 6.65)	< 0.001	2.81 (2.11, 3.76)	< 0.001
**Family member addiction^m^**								
No	1.00 [Reference]		1.00 [Reference]		1.00 [Reference]		1.00 [Reference]	
Yes	2.38 (1.86, 3.04)	< 0.001	1.41 (0.81, 2.43)	0.22	2.09 (1.14, 3.83)	0.02	1.83 (1.36, 2.47)	< 0.001

ADHD, attention deficit hyperactivity disorder; ASD, autism spectrum disorder; ID, intellectual disorder; LD, learning disorder.

^a^Weighted multivariable logistic regression models to evaluate associations of the prevalence of ADHD, ASD, ID, and LD with demographics, personal experience, comorbidities or psychological disorders, and household or parental factors.

^b^Adjusting age, sex, race, health insurance, urbanicity, and geographic regions in each model.

^c^Individuals are considered currently insured if they currently have coverage through private health insurance, Medicare, Medicaid, Children’s Health Insurance Program (CHIP), military [TRICARE, Veterans Administration (VA), and CHAMP-VA], other state-sponsored health plans, or other government programs. Individuals without any of the aforementioned coverages or with only Indian Health Service coverage or a non-comprehensive plan that covers only dental, vision, or prescription drugs are considered uninsured.

^d^Urban area stands for large central metro, large fringe metro, medium metro, and small metro.

^e^Individuals are considered to have anxiety symptoms if they seemed anxious, nervous, or worried equal to or more than a few times a year.

^f^Individuals are considered to have depression symptoms if they seemed sad or depressed equal to or more than a few times a year.

^g^Other means cohabiting parents residing in the same household as sample children or at least one related or unrelated adult (not a parent) in the same household as sample children.

^h^The highest level of education refers to the highest level of education among all sample child’s parents.

^i^Individuals are considered to live with the elderly when there is at least one person aged over 65 in the family.

^j^Individuals are considered being bullied when parents are certainly true or somewhat true that children have been picked on or bullied by others in the last 6 months.

^k^Individuals are considered to have a family member who was incarcerated when they had ever lived with a parent who was incarcerated.

^l^Individuals are considered to have a family member who was mentally ill when they had ever lived with anyone mentally ill/severely depressed.

^m^Individuals are considered to have a family member with addiction problems when they had ever lived with anyone with alcohol/drug problems.

## Discussion

Based on the counts of currently having NDDs from NHIS in 2019 and 2020, we estimated that the prevalence of ADHD, ASD, ID, and LD was 8.5, 2.9, 1.4, and 6.4%, respectively. Our estimates on NDDs prevalence were lower than previous estimates, where cases were counted by ever had NDDs in the numerator calculation (Zablotsky et al., 2019b; Zablotsky and Black, 2020; Khan et al., 2021; Yang et al., 2021). Since NDDs symptoms could alleviate through behavioral and educational therapies or after maturation (Magiati et al., 2014), the prevalence might be overestimated if counting all with historical NDDs (Kogan et al., 2018; Xu et al., 2018; Zablotsky et al., 2019a). On the other hand, the prevalence estimates of ADHD and ASD have been continuously growing in recent decades (Visser et al., 2014; Zablotsky et al., 2019a; Chiarotti and Venerosi, 2020). On the other hand, our results are higher than those in earlier studies. In the US, the prevalence estimates of ADHD and ASD have been continuously growing (Visser et al., 2014; Zablotsky et al., 2019a; Chiarotti and Venerosi, 2020). The reasons could be in part explained by the modified diagnosis criteria and the increase in health awareness (American Psychiatric Association, 2013; Polanczyk et al., 2014). The difference in data collection may also affect the prevalence estimation. Studies that used hospital-reported prescription data may underestimate the prevalence estimates due to missing part of children who lack medical support or were not suitable for the prescription because medications are not always required in the intervention and treatment for NDDs (Cairncross and Miller, 2020). With the new diagnosis standards, our estimates might be closer to the true prevalence among US children and adolescents. In addition, we further estimated the prevalent differentiation by demographics, parental characteristics, comorbidities/mental problems, as well as household and stressful life events, which led to more clues about susceptible populations or risk factors.

In line with previous studies, we found a significant difference in the NDDs prevalence by sex (Thapar et al., 2017). In our study, boys are more likely to have ADHD, ASD, ID, and LD than girls. There could be a phenotypic difference in the relevant symptoms between boys and girls, while they have the same diagnostic standard. The plausible explanation for the sex difference could be relevant symptoms in boys can be onset earlier and are less likely to be concealed compared with girls because of less externalizing behavior in girls and potential chromosome-linked genetic difference (Thapar et al., 2017; Mowlem et al., 2019; Dillon et al., 2021; Walsh et al., 2021). More studies are expected to enrich the mechanism explanation. Furthermore, ADHD and LD could be initiated based on cumulative effects of childhood-onset negative events; hence, the related symptoms could appear at an older age in children and adolescents (Arora et al., 2018). The coverage of public health insurance can raise parents’ concern for the early growth of children and thus improve the detection rate of NDDs, which is consistent with previous findings (Danielson et al., 2018).

Evidence showed that NDDs are frequently comorbid with other psychiatric disorders and medical conditions. Although there are some overlapping symptoms among these NDDs, the current diagnostic procedure is mainly based on the Diagnostic and Statistical Manual of Mental Disorders, fifth edition (DSM-5) (American Psychiatric Association). There are some gold-standard diagnostic measures for NDDs, such as the Autism Diagnostic Interview-Revised and the Autism Diagnostic Observation Schedule, the second edition for ASD and the standardized ADHD rating scales, structured interviews such as the KSADS-PL, and behavioral observations for ADHD. The diagnostic procedure of DSM-5 also emphasizes the core symptoms and distinguishes different disorders by specific scales or based on parents’ and teachers’ reports on children’s behavior (American Psychiatric Association; Nickel et al., 2019). Prevalence estimates of ADHD, ASD, ID, and LD were found higher in individuals with depressive and anxiety symptoms or individuals living with anyone mentally ill, suggesting hereditation of psychiatric disorders across generations, which is parallel with previous findings (Chandra et al., 2016). However, since psychiatric symptoms are highly heritable, further studies are warranted to investigate if parental psychiatric care can bring a positive influence on the alleviation of psychiatric severity of the next generation. The observed relationship between asthma and ADHD in our study is consistent with the previous investigation (Zheng et al., 2016; Cortese et al., 2018; Kim et al., 2020), and the relationship between asthma and LD is also significant. Effects of allergic inflammatory cytokines and elevated psychological stress were broadly recognized as contributors to the neuro-immunological and psychological mechanisms between asthma and NDDs (Lord et al., 2018; Papi et al., 2018; Kim et al., 2020; Posner et al., 2020). In addition, the stress increased by NDDs may contribute to poor adherence to treatment, and worse asthma control, thus raising more stress (Papi et al., 2018). Such vicious circles may also apply to NDDs treatments as well as raise the anxiety and depression levels of children.

Living with married parents and other children may be associated with a lower chance of NDD symptoms among US children and adolescents. The reason could be family companionship and motivation which bring positive effects on the sense of achievement and mental development for individuals (Karst and Van Hecke, 2012; Deater-Deckard, 2017). It might be also related to the higher income in the family with married parents and more children (Lord et al., 2018; Posner et al., 2020). It is of note that observations in the cross-sectional study might not build the causal relationship between family/sibling accompany and the onset of NDDs. Reverse causality is also likely since parents may pay more attention to children already with NDD symptoms. In addition, having a child with NDDs may contribute to a higher rate of divorces due to parenting stress. More epidemiological evidence is highly demanded in further studies. Children and adolescents in low-income families were more likely to have NDDs, which is in line with the observation that cases of NDDs were less prevalent among those living in houses owned by their families than their counterparts. Lower family income may cause less companionship from working parents and a lack of medical care or early intervention before diagnosis. Thus, a lack of financial support would cause a higher prevalence of NDDs and reduce the chance of adulthood independence and symptoms decreasing (Lord et al., 2018; Posner et al., 2020; Zuvekas et al., 2021). Moreover, although house ownership is entangled with the family’s economic level, a stable living environment should be valuable in the neurodevelopment of children.

We found that victims of/witnessed violence or those living with anyone ever incarcerated were associated with a higher prevalence of ADHD, ID, and LD. It might be partly explained by genetic factors as violent family members may also suffer from NDDs. On the other hand, experiencing violent crime may cause disrupted cortisol patterns among children, which may increase their stress and lead to mental health problems (Heissel et al., 2018). Moreover, aggression or defensiveness in daily interactions due to heightening stress may obstruct getting support from communities and society, and thus, accelerate the deterioration of neurodevelopmental or psychiatric disorders (Cuartas and Leventhal, 2020). Parents with alcohol or drug problems may be associated with the initiation of ADHD, ID, and LD in children and adolescents. Most parents with serious alcohol/drug problems may also misuse alcohol or drugs during pregnancy or breastfeeding. The cognition and mental health of offspring might be influenced by parental drinking/drug use in sensitive periods though mechanisms remain unclear (Gibson and Porter, 2018; Syed and Gilbert, 2019). In addition, because of the inequality of income or other reasons, bullying is a prevalent and preventable risk factor for children and adolescence (Elgar et al., 2019; Zhang et al., 2020). In our study, experiencing bully was associated with the prevalence of ADHD, ASD, ID, and LD. Based on the risk of long-term emotional and behavioral problems due to posttraumatic stress disorder, experiencing bullying may have effects on the progress of ASD rather than initiation because most ASD is detected before school age (Lai et al., 2014).

The findings in our study can be interpreted to represent national estimates among current US children and adolescents because of the large sample size, high response rate, and systematic weighting procedure of NHIS in 2019 and 2020. There are several limitations of the study. First, information on diagnosed NDDs was collected by parent reporting rather than clinical evaluation or educational records. Although non-differential misclassification is inevitable due to recall bias or conceptual confusion, previous evidence showed a high concordance rate (93–98%) between parent reports (such as ASD) and clinician’s diagnosis in verbal children (Daniels et al., 2012), which may reflect the validation of parent reports. Second, the prevalence estimates in those without healthcare insurance might be unreliable due to the high relative standard error (>30%). The sample size for specific subgroups could be deficient because only NHIS in 2019 was leveraged in our study. Prevalence estimates across multiple years are in need to generate results with higher reliability. Third, non-response bias and selection bias were also likely that families without a settled household or who tend to ignore phone calls can result in a lower response. Moreover, the COVID-19 pandemic in 2020 led to a smaller sample size than in previous years and a change of investigation method from mainly in-person interviews to phone interviews. However, the survey attempted to mitigate any differential non-response and selection bias by non-response weighting adjustments and post-stratification adjustments (National Center for Health Statistics, 2021).

## Conclusion

Knowledge about the national-wide prevalence of NDDs serves for the assessment of disease burden and informing the healthcare management strategies. Our study updated nationwide estimates for the prevalence of ADHD, ASD, ID, and LD among US children and adolescents aged 3–17 years in 2019 and 2020, and also detected the stratification among subgroups of demographics, comorbidity/mental problems, household/parental characteristics, and stressful life events. Future studies are in demand to explore the risk factors and corresponding bio-mechanisms for the onset and progression of NDDs, especially ADHD and ASD.

## Data availability statement

Publicly available datasets were analyzed in this study. This data can be found here: https://www.cdc.gov/nchs/nhis/data-questionnaires-documentation.htm.

## Author contributions

JR conceived the idea and designed the study. YY and MZ acquired the data. SZ and JR cleaned and analyzed the data. YY and LH interpreted the results. YY, LH, and JR drafted the manuscript. JR, LH, SZ, MX, JZ, SC, and HW revised the manuscript. All authors contributed to the content and critical revision of the manuscript and approved the final version.
